# Tiny Lungs, Big Decisions: A Meta-Analysis Comparing Minimally Invasive Surfactant Therapy Versus Intubation–Surfactant–Extubation in Preterm Neonates With Respiratory Distress Syndrome

**DOI:** 10.1155/ijpe/8045343

**Published:** 2025-08-14

**Authors:** Flavio Veintemilla-Burgos, Geovanna Minchalo-Ochoa, Sebastian Balda, Ivo Diaz-Djevoich, Rodolfo Kronfle, Matias Panchana-Lascano, Thomas Leone-Berry

**Affiliations:** ^1^Faculty of Medical Sciences, Universidad Católica Santiago de Guayaquil, Guayaquil, Ecuador; ^2^Summa Veritas Medical Research, Hospital Clinica Kennedy, Guayaquil, Ecuador

**Keywords:** INSURE, MIST, premature infant, respiratory distress syndrome, surfactant

## Abstract

**Background:** Neonatal respiratory distress syndrome (NRDS) is a leading cause of morbidity and mortality in preterm infants. Minimally invasive surfactant therapy (MIST) has emerged as a promising alternative to traditional approaches, aiming to reduce mechanical ventilation while maintaining spontaneous breathing. This meta-analysis compares the efficacy and safety of MIST versus the intubation–surfactant–extubation (INSURE) method in preterm neonates with NRDS.

**Methods:** We searched PubMed, Scopus, and Embase for eligible studies. Two independent reviewers screened studies via the Rayyan platform. We included randomized controlled trials and observational cohorts of preterm neonates (< 37 weeks) with NRDS requiring surfactant therapy, comparing MIST and INSURE. Data extraction included study characteristics, demographics, and clinical outcomes. Risk of bias was assessed using the Newcastle–Ottawa Scale and Cochrane tools. Risk ratios (RRs) and mean differences (MDs) with 95% confidence intervals (CIs) were calculated using a random-effects model. Heterogeneity was evaluated via *I*^2^ statistics.

**Results:** Then, 17 studies (*n* = 1931 neonates; MIST: 913, INSURE: 932) were included. Baseline characteristics were similar between groups. Mortality did not differ significantly (RR 0.62; 95% CI 0.38–1.01; *p* = 0.05). MIST was associated with reduced risks of bronchopulmonary dysplasia (RR 0.59; 95% CI 0.45–0.76), intraventricular hemorrhage (RR 0.66; 95% CI 0.48–0.92), patent ductus arteriosus (RR 0.75; 95% CI 0.61–0.93), and pneumothorax (RR 0.54; 95% CI 0.34–0.87). Rates of pulmonary hemorrhage and surfactant reflux were comparable. MIST also resulted in shorter oxygen day requirements (MD −2.45; *p* = 0.04) and need for mechanical ventilation (RR 0.54; *p* = 0.002). Duration of ventilation and NICU showed no significant differences.

**Conclusion:** MIST proved to be a safer and more effective alternative to INSURE in preterm infants with NRDS, reducing several complications and mechanical ventilation needs. These findings highlight MIST's potential as a preferred approach, warranting further research to support broader implementation.

## 1. Background

Neonatal respiratory distress syndrome (NRDS) remains a leading cause of morbidity and mortality among preterm infants [1]. While traditional surfactant therapy via endotracheal intubation followed by mechanical ventilation has improved outcomes, this is an approach linked to complications, including bronchopulmonary dysplasia (BPD). In response, minimally invasive surfactant therapy (MIST) has emerged as an alternative, designed to deliver surfactant while minimizing these complications by preserving spontaneous breathing [2].

Several studies have compared MIST to the conventional intubation–surfactant–extubation (INSURE) technique, suggesting benefits of MIST in reducing the need for mechanical ventilation and incidence of BPD. However, existing literature presents several limitations that hinder definitive conclusions. Many studies exhibit heterogeneity in patient populations, gestational ages, and definitions of outcomes, making comparisons challenging. Additionally, variations in the timing of surfactant administration and the criteria for initiating mechanical ventilation contribute to inconsistent findings. For instance, a retrospective study involving infants with very low birth weight (< 1500 g) demonstrated that MIST was associated with a lower incidence of death or BPD, reduced duration of oxygen therapy, and fewer instances requiring mechanical ventilation compared to the INSURE approach [3]. Furthermore, a meta-narrative review also supports the potential benefits of MIST, reporting a decreased requirement for mechanical ventilation within 72 h of life compared to standard care; however, no significant difference was found in the incidence of BPD [4].

Although existing studies suggest favorable outcomes associated with MIST, substantial heterogeneity in study designs, methodological rigor, and outcome definitions limits the ability to draw definitive conclusions. Notably, there is a lack of large-scale, randomized controlled trials (RCTs) directly comparing MIST and INSURE methods across diverse clinical settings. These gaps underscore the need for a comprehensive meta-analysis to synthesize available evidence and provide clearer guidance on the efficacy and safety of MIST versus ENSURE in preterm infants with NRDS. This meta-analysis is aimed at evaluating and comparing the efficacy and safety of MIST and INSURE, focusing on key outcomes such as mortality rate, need for oxygen, need for mechanical ventilation, requirement for invasive ventilation within 72 h of surfactant administration, need for intubation, and secondary outcomes in this population. The results of this study will provide valuable insights to inform clinical decision-making and guide future research in neonatal respiratory care.

## 2. Materials and Methods

### 2.1. Literature Search

A comprehensive and detailed literature search was conducted across PubMed, Scopus, and Embase databases on March 25, 2025. The relevant studies that met our inclusion and exclusion criteria were selected. The search terms used were as follows: “(intratracheal OR invasive OR Insure OR intubation surfactant extubation OR intubation-surfactant-extubation) AND (MIST OR minimally invasive surfactant therapy) AND surfactant.” This meta-analysis was performed in accordance with the PRISMA 2020 guidelines (Table S1, digital content).

### 2.2. Inclusion and Exclusion Criteria

The Rayyan platform was used for screening and selecting eligible studies. Two co-authors independently conducted the initial screening based on titles and abstracts, with any selection conflicts resolved by a third reviewer.

The inclusion criteria comprised premature neonates (< 37 weeks) diagnosed with NRDS and requiring surfactant therapy. The selected studies included observational cohort studies and RCTs comparing MIST and INSURE, provided they reported at least one of the predefined primary outcomes. Abstracts were also considered if they contained sufficient data.

The exclusion criteria included systematic reviews, meta-analyses, narrative reviews, case reports/series, editorials, study protocols, commentaries, letters, and studies published in languages other than English. Additionally, term neonates and those with congenital anomalies affecting the respiratory system were also excluded.

### 2.3. Data Extraction and Quality Assessment

A data extraction sheet was elaborated using Google Sheets by one co-author. Of the included articles, data was extracted independently by coauthors, with approximately three articles assigned to each. The data collected involved first author last name, the year of publication, location where the study was conducted, the study center, the study design and duration, the number of patients, and for how long they were followed up, the population's characteristics, number of patients, sex, gestational age in weeks, birth weight, whether the mother received corticosteroids, presence of gestational diabetes mellitus, premature rupture of membranes, severity of RDS/RDS SCORE, cesarean section, and APGAR scores for 1 and 5 min. Collected outcomes included mortality rate, need for oxygen, need for mechanical ventilation or intubation, and complications such as pulmonary hemorrhage, patent ductus arteriosus, pneumothorax, BPD, intraventricular hemorrhage (IVH), and drug/surfactant reflux. Continuous outcomes comprised duration of NICU stay/hospitalization, duration of intubation, and need for oxygen in days.

The datasets used and/or analyzed during this meta-analysis are available from the corresponding author upon reasonable request. Data will be shared in the deidentified form, along with the search strategies, study protocols, and statistical codes used for the analyses. Access is subject to a data-sharing agreement.

Two independent co-authors assessed each study for risk of bias using the Newcastle–Ottawa Scale for the five observational studies included (Table S2, digital content) and the Cochrane Risk of Bias tool for the 12 RCT studies. The assessment of the deviations from intended interventions, bias domains, was unclear due to the lack of blinding of clinicians, as well as measurement of outcomes in some studies (Table S3, digital content).

### 2.4. Outcome Definition Standardization

Variability in outcome definitions was observed across the included studies, particularly for BPD, IVH, and patent ductus arteriosus. To address this issue, outcomes were extracted and categorized using clinically comparable definitions to maintain consistency. For instance, BPD was included regardless of whether it was defined by oxygen requirement at 28 days of life or at 36 weeks postmenstrual age, as both criteria represent the same underlying pathology and are diagnosed in the same manner. Similarly, IVH was considered for inclusion when diagnosed by cranial ultrasound. PDA was included when identified through echocardiography and clinical signs. Studies that had unclear or incompatible outcome definitions were excluded from pooled analysis for the respective variables. In cases where definitions were not explicitly stated, we reviewed the methods section of the study and their descriptions to confirm clinical equivalence. This approach enabled standardized outcome data extraction while considering the heterogeneity among the included studies.

### 2.5. Statistical Analysis

For the study, statistical analysis risk ratios (RRs) with 95% confidence intervals (CIs) for dichotomous outcomes, as well as mean differences (MDs) with 95% CIs for continuous outcomes, were calculated. A *p* value of less than 0.05 was considered to be statistically significant. Heterogeneity between the studies was assessed using *I*^2^ statistics, with *I*^2^ > 50% indicating substantial heterogeneity. In addition, meta-regression analyses were performed with birth weight and mean gestational age as continuous moderators to examine their influence on effect sizes for key outcomes. Potential publication bias was evaluated using Egger's test for small-study effects, and none of the outcomes indicated evidence of bias. Funnel plots for each of the outcomes were analyzed and appeared symmetric, which further supports the absence of substantial publication bias. Statistical analyses, including figures, were performed using Stata 18 (2023) and with Review Manager (RevMan 5). Rayyan was used for the screening process.

## 3. Results

**Figure 1:**
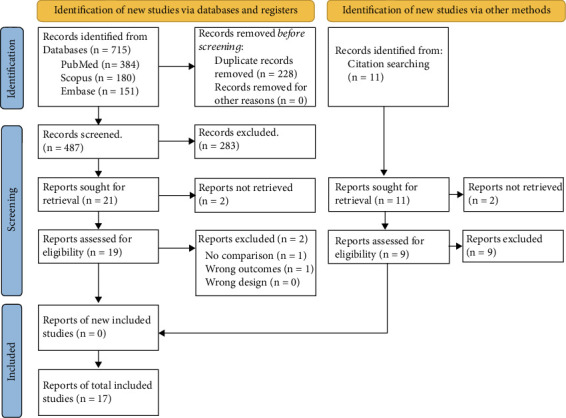
PRISMA flowchart with search strategy used in the study.

Then, 17 studies were included for the final analysis after applying our inclusion and exclusion criteria. The disposition is 13 RCTs, 2 prospective cohorts, and 2 retrospective cohorts. A complete search strategy is shown in Figure 1, and a summary of the studies' characteristics is shown in Table 1. A total of 1931 subjects were included, from which 913 were part of the MIST group, and 932 in INSURE. The average male patients in the MIST group corresponds to 54.5%, with a mean gestational age (weeks) of 30.4 ± 1.3, similar to the neonates in the INSURE group, with 53.7% of neonates being males, with a mean gestational age of 30.2 ± 1.4. A detailed description of baseline characteristics of the sample populations is illustrated in Table 2.

### 3.1. Assessment of Mortality and Complications

**Figure 2:**
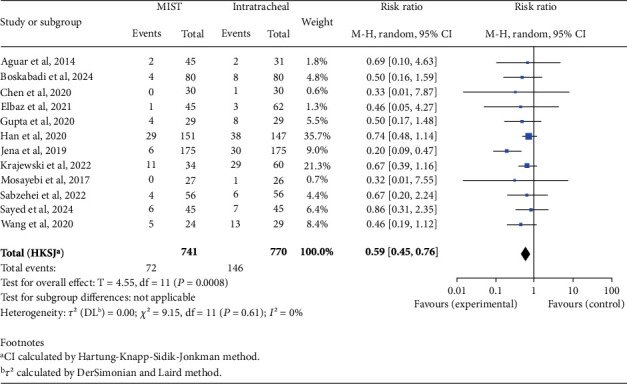
Bronchopulmonary dysplasia. Forest plot using relative risk of bronchopulmonary dysplasia after administering surfactant via MIST versus intratracheal instillation. Statistics of a test of heterogeneity among the studies are also shown. CI, confidence interval; MIST, minimally invasive surfactant therapy.

**Figure 3:**
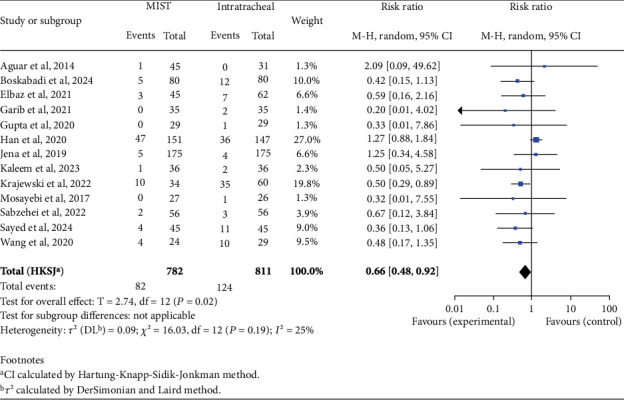
Intraventricular hemorrhage. Forest plot using relative risk of intraventricular hemorrhage after administering surfactant via MIST versus intratracheal instillation. Statistics of a test of heterogeneity among the studies are also shown. CI, confidence interval; MIST, minimally invasive surfactant therapy.

**Figure 4:**
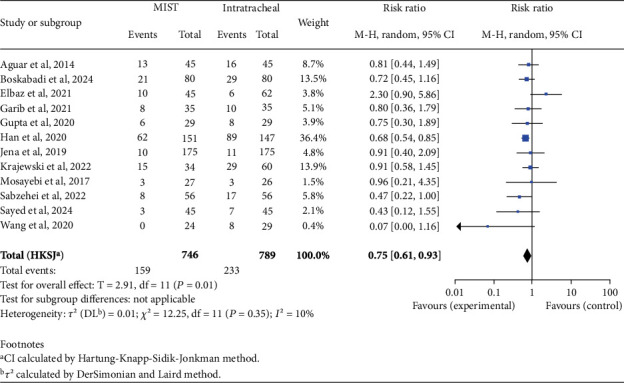
Persistent ductus arteriosus. Forest plot using relative risk of persistent ductus arteriosus after administering surfactant via MIST versus intratracheal instillation. Statistics of a test of heterogeneity among the studies are also shown. CI, confidence interval; MIST, minimally invasive surfactant therapy.

**Figure 5:**
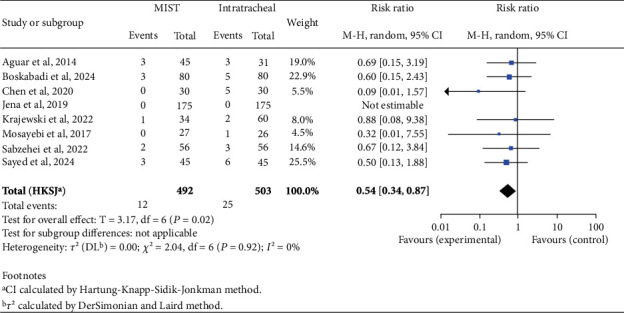
Pneumothorax. Forest plot using relative risk of pneumothorax after administering surfactant via MIST versus intratracheal instillation. Statistics of a test of heterogeneity among the studies are also shown. CI, confidence interval; MIST, minimally invasive surfactant therapy.

There was no statistically significant difference in mortality rates between the two groups (RR 0.62; 95% CI 0.38–1.01; *p* = 0.05). When analyzing complications, we found an increased risk in the INSURE group for BPD (RR 0.59; 95% CI 0.45–0.76; *p* < 0.001), PVA (RR 0.66; 95% CI 0.48–0.92; *p* = 0.02), patent ductus arteriosus (PDA) (RR 0.75; 95% CI 0.61–0.93; *p* = 0.01), and pneumothorax (RR 0.54; 95% CI 0.34–0.87; *p* = 0.02), as shown in Figures 2, 3, 4, and 5. There was no significant difference in the incidence of pulmonary hemorrhage (RR 0.69; 95% CI 0.38–1.25; *p* = 0.18) or surfactant reflux (RR 1.47; 95% CI 0.71–3.03; *p* = 0.22).

### 3.2. Assessment of Secondary Outcomes

**Figure 6:**
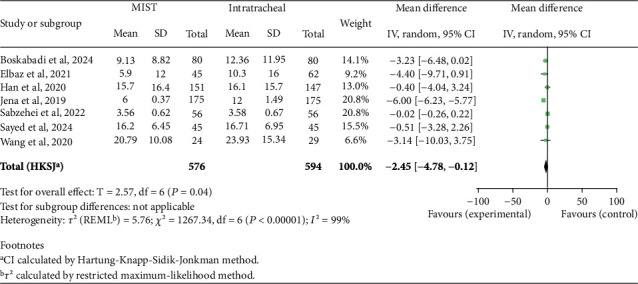
Days needing oxygen. Forest plot using mean difference in days needing oxygen after administering surfactant via MIST versus intratracheal instillation. Statistics of a test of heterogeneity among the studies are also shown. CI, confidence interval; MIST, minimally invasive surfactant therapy.

**Figure 7:**
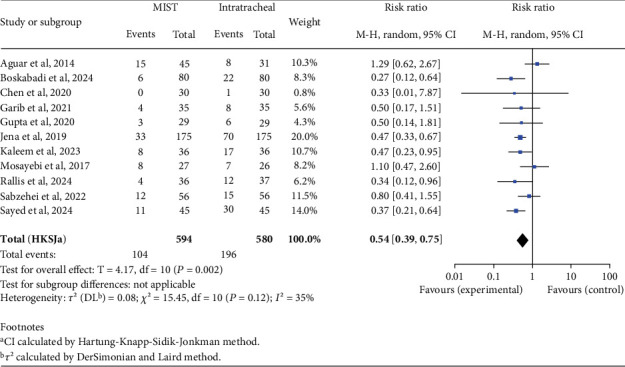
Need for mechanical ventilation. Forest plot using relative risk of requiring mechanical ventilation after administering surfactant via MIST versus intratracheal instillation. Statistics of a test of heterogeneity among the studies are also shown. CI, confidence interval; MIST, minimally invasive surfactant therapy.

Regarding the number of days requiring oxygen, we found a statistically significant MD favoring MIST (MD −2.45; 95% CI −4.78 to −0.12; *p* = 0.04), indicating that MIST patients have a shorter duration of days requiring oxygen during hospitalization. We also found a statistically significant reduced risk for needing mechanical ventilation in the MIST group (RR 0.54; 95% CI 0.39–0.75; *p* = 0.002), as shown in Figures 6 and 7. In contrast, we did not observe a significant MD in the duration of mechanical ventilation (MD −1.83; 95% CI −6.15 to 2.50; *p* = 0.34) or length of NICU stay (MD −4.08; 95% CI −8.89 to 0.83; *p* = 0.09) between the two groups.

### 3.3. Meta-Regression Analysis

Meta-regression analyses did not reveal any significant associations between mean gestational age or birth weight and the outcomes examined. For example, mortality was not significantly associated with gestational age (*β* = −0.019; 95% CI −0.66–0.62; *p* = 0.953) or birth weight (*β* = 0.002; 95% CI: −0.0007–0.004; *p* = 0.171). Similarly, no significant effects were observed for BPD or patent ductus arteriosus. A summary of meta-regression results for all other outcomes, including intraventricular hemorrhage and pneumothorax, is provided in Table 3.

### 3.4. Sensitivity Analysis and Publication Bias

In the sensitivity analysis for the different complications, this study found that the statistical significance of certain outcomes was influenced by individual studies. For intraventricular hemorrhage, the removal of Krajeski et al. [13] rendered the result nonsignificant (RR 0.73; 95% CI 0.50–1.06; *p* = 0.06). Similarly, for patent ductus arteriosus, excluding Han et al. [10] resulted in a nonsignificant finding (RR 0.83; 95% CI 0.60–1.06; *p* = 0.11). Lastly, in the analysis of days needing oxygen, removing Jena et al. [11] led to a loss of statistical significance (MD −0.97; 95% CI−2.64 to 0.70; *p* = 0.19). In all cases, these studies had large sample sizes, contributing significantly to the overall weight of the analysis. However, the general trend remained consistent across the remaining studies, suggesting that the observed effects were not driven by a single study but rather influenced by statistical power.

Egger's test for small-study effects did not indicate significant publication bias for any of the analyzed outcomes. The results were as follows: BPD (*β* = −0.55, SE = 0.583, *z* = −0.95, *p* = 0.3417), intraventricular hemorrhage (*β* = 0.37, SE = 0.675, *z* = 0.55, *p* = 0.5795), PDA (*β* = 0.05, SE = 0.522, *z* = 0.10, *p* = 0.9210), days needing oxygen (*β* = 0.03, SE = 1.060, *z* = 0.02, *p* = 0.9805), and need for mechanical ventilation (*β* = −0.15, SE = 0.932, *z* = −0.16, *p* = 0.8725). Funnel plots were visually inspected and appeared symmetric, further supporting the absence of substantial publication bias. These plots are presented in the Supporting Information (Figures S1–S6, digital content).

### 3.5. Certainty Assessment

In order to analyze the certainty of evidence, we conducted an assessment with the GRADE scoring system (Table S4, digital content). Outcomes such as reduction in BPD, pneumothorax, and need for mechanical ventilation were supported by high-certainty evidence, reflecting consistent and precise findings across multiple RCTs. Moderate certainty was assigned to mortality, PDA, IVH, and days requiring oxygen due to borderline statistical significance or sensitivity to single-study effects. Outcomes like pulmonary hemorrhage, surfactant reflux, and NICU stay had low certainty due to wide CIs and inconsistent findings.

## 4. Discussion

This study highlights several factors supporting the use of MIST over the standardized INSURE method to treat preterm infants with NRDS, offering a safer alternative to surfactant delivery by significantly reducing overall complications.

By minimizing invasive procedures, MIST promotes hemodynamic stability and reduces the risk of cardiovascular complications such as PDA and IVH. The findings are consistent with prior research demonstrating that MIST is associated with a lower risk of cardiovascular complications compared to INSURE [20]. Avoidance of endotracheal intubation reduces fluctuations of intrathoracic pressure and cerebral blood flow, a key factor in IVH [21]. Similarly, the positive pressure of ventilation could interfere with normal closure of the ductus arteriosus [22]. PDA is one of the most common complications in neonates, often resulting in hemodynamic instability and, in some cases, requiring surgical intervention [23]. Likewise, IVH may lead to long-term neurodevelopmental impairment, further emphasizing the importance of effective management strategies [24]. By reducing these risks, MIST contributes to improved short-term outcomes and potentially decreases long-term morbidity.

Regarding respiratory outcomes, this study highlights the advantages of using MIST rather than INSURE. Specifically, the need for mechanical ventilation, which is a major risk factor for BPD was significantly lower in the MIST group. Furthermore, a lower incidence of BPD can be extrapolated to a lower incidence of pneumothorax. An explanation could be that the minimally invasive nature of MIST allows it to administer surfactant while maintaining spontaneous breathing, which reduces the need for positive pressure ventilation and may explain the lower incidence of complications associated with mechanical ventilation and barotrauma [25]. The preservation of spontaneous breathing also promotes greater physiological lung expansion, which reduces the risk of alveolar overdistension and lung injury [26].

Finally, the MIST group required a shorter duration of oxygen supplementation. This could be explained as MIST promotes more effective surfactant distribution and alveolar recruitment, resulting in improved oxygenation [27]. Prolonged oxygen dependency is associated with complications such as retinopathy of prematurity and chronic lung disease [28, 29]. Therefore, a faster withdrawal from oxygen support may contribute to improved recovery and reduced morbidity.

Although the results of this meta-analysis found clear benefits favoring MIST, limitations must be acknowledged. Among these are methodological heterogeneity, including variations in surfactant administration techniques and patient selection criteria. Additionally, outcomes such as mortality and surfactant reflux did not demonstrate statistically significant differences, which highlights the need for further high-quality trials.

Despite these promising findings, it is essential to interpret the results of this meta-analysis with caution. Many of the included studies had varying degrees of methodological quality, and a considerable number were assessed as having a high or unclear risk of bias in at least one domain. This may weaken the strength of the conclusions drawn. Biases such as lack of blinding, inconsistent outcome definitions, or selective reporting could potentially overestimate the benefits of MIST or underestimate the risks associated with either intervention. Despite attempts to harmonize outcome definitions across studies, existing variability in diagnostic criteria may have contributed to some degree of heterogeneity in our results. Moreover, variability in surfactant administration techniques and patient characteristics across studies may introduce further heterogeneity. To strengthen the evidence base, future studies should employ standardized protocols, ensure rigorous methodology, and focus on reducing bias through appropriate randomization, blinding, and complete outcome reporting.

## 5. Conclusions

In conclusion, our findings support MIST as a more effective and safer alternative than INSURE for preterm infants with NRDS, showing benefits that include reduced complications and lower rates of mechanical ventilation. Most noticeable is the decrease in overall complications with the MIST intervention. This study exemplifies the potential of MIST as an upcoming treatment option. While MIST has the potential to become the new standard of care, further research is necessary to address implementation barriers and optimize its use in different clinical scenarios.

## Figures and Tables

**Table 1:** Characteristics of included studies. MIST, minimally invasive surfactant therapy; RDS, respiratory distress syndrome; INSURE, intubation, surfactant, extubation; CPAP, continuous positive airway pressure; NICU, neonatal intensive care unit; IMV, invasive mechanical ventilation; BPD, bronchopulmonary dysplasia; PDA, patent ductus arteriosus; NIPPV, nasal intermittent positive pressure ventilation; LISA, less invasive surfactant administration; PIVH, periventricular-intraventricular hemorrhage; VLBW, very low birth weight.

**Study ID**	**Location**	**Duration**	**Design**	**No. of patients**		**Study conclusions**
				**MIST**	**INSURE**	
Aguar et al. (2014) [5]	University and Polytechnic Hospital La Fe. Valencia, Spain	2011–2012	Prospective cohort	45	31	Surfactant administration using MIST is feasible in preterm infants with RDS. No significant differences in secondary respiratory outcomes were found between the MIST and INSURE techniques.
Boskabadi et al. (2024) [6]	Qaem (AS) and Imam Reza (AS) of Mashhad University of Medical Sciences. Mashad, Iran	2022–2023	Randomized controlled trial	80	80	MIST reduces the need for reintubation, the duration of oxygen therapy, and the duration of CPAP requirement, without increasing the incidence of prematurity-related complications compared to the standard INSURE method
Chen et al. (2020) [7]	First Affiliated Hospital of Bengu Medical Collage. Anhui, China	2018–2019	Randomized controlled trial	30	30	MIST can significantly improve oxygen metabolism, reduce secondary surfactant application and pneumothorax rates, shorten hospitalization time, and improve the prognosis of premature infants with respiratory distress syndrome (RDS)
Elbaz et al. (2021) [8]	Ziv Medical Center. Tsfat, Israel	2012–2017	Retrospective cohort	45	62	Transition to MIST was associated with significantly reduced need for oxygen, mechanical ventilation and surfactant, and a borderline shortened NICU admission.
Garib et al. (2021) [9]	Menoufia Hospital. Menoufia, Egypt	No dates reported	Randomized controlled trial	35	35	The MIST method was the most successful with a rate of 96.5% in terms of time and the child's response to treatment. However duration of invasive mechanical ventilation were higher in MIST group than InSurE group.
Gupta et al. (2020) [2]	SSKM Hospital Kolkata West Bengal India. Kolkata, India	2019	Randomized controlled trial	29	29	There is no difference between MIST and InSurE in preterm neonates with RDS with NIPPV as a primary mode of respiratory support
Han et al. (2020) [10]	Multicenter. Hebei, China	2017–2018	Randomized controlled trial	151	147	MISA had no clear benefit on the incidence of BPD, but it was related to a reduction in PDA. It is an appropriate therapy for spontaneous breathing in infants with extremely low birth weight and NRDS.
Jena et al. (2019) [11]	Multicenter. India	2013–2017	Randomized controlled trial	175	175	In preterm neonates with RDS who are stabilized on CPAP, the SurE technique for surfactant delivery results in a reduced need for MV and also may decrease the rate of BPD
Kaleem et al. (2023) [12]	Children's Hospital and Institute of Child Health. Lahore, Pakistan	2021–2022	Randomized controlled trial	36	36	Surfactant therapy through MIST is effective and there is significantly reduced need of IMV than in INSURE.
Krajewski et al. (2022) [13]	Medical University of Warsaw. Warsaw, Poland	2009–2013	Retrospective cohort	34	60	Noninvasive methods of surfactant administration, such as with the LISA and INSURE methods, are safe and effective in the treatment of respiratory distress syndrome, with no increase in the rate of pneumothorax.
Manuela et al. (2017) [14]	County Emergency Hospital Tîrgu Mureș. Trasgu Mures, Rumania	No dates reported	Randomized controlled trial	17	9	MIST is a safe and potentially effective alternative to other techniques of surfactant administration
Mosayebi et al. (2017) [15]	Roointan-Arash Maternity Hospital. Tehran, Iran	2013–2014	Randomized controlled trial	27	26	Surfactant administration via a thin catheter is a feasible and effective treatment for preterm infants.
Rallis et al. (2024) [16]	No location reported	No dates reported	Prospective cohort	36	37	MIST technique was associated with a lower need for intubation within 72 h of age, but with no significant differences regarding BPD or other neonatal morbidities
Sabzehei et al. (2022) [17]	Hamadan University of Medical Sciences. Hamdan, Iran	2019–2020	Randomized controlled trial	56	56	Surfactant administration using MIST could be a good replacement for INSURE in preterm infants with RDS.
Sayed et al. (2024) [18]	Assiut University Children Hospital. Assiut, Egypt	2024	Randomized controlled trial	45	45	MIST technique reduces the need for MV and may also lower the rate of PIVH in certain susceptible subgroups
Wang et al. (2020) [3]	Changhua Christian Children's Hospital, Chung-Shan Medical University Hospital. China	2015–2018	Retrospective cohort	24	29	MIST reduce the duration of mechanical ventilation and may reduce the composite outcome of death or BPD for VLBW infants with RDS.
Zhao et al. (2020) [19]	China. No other location reported	2017 - 2019	Randomized controlled trial	48	45	MIST is effective in the treatment of RDS in moderately preterm infants, but it presents not too much benefits of effectiveness and complications, as well as the disadvantage of drug reflux

**Table 2:** Baseline characteristics of patients included in the studies. MIST, minimally invasive surfactant therapy; INSURE, intubation, surfactant, extubation; SD, standard deviation.

**Study ID**	**No. of patients**		**Male sex (%)**		**Gestational age (** **m** **e** **a** **n** **weeks** ± **SD)**		**Birth weight (** **m** **e** **a** **n** ± **SD)**		**Corticosteroid (%)**	
	**MIST**	**INSURE**	**MIST**	**INSURE**	**MIST**	**INSURE**	**MIST**	**INSURE**	**MIST**	**INSURE**
Aguar et al. (2014) [5]	45	31	22 (48.9)	16 (51.6)	30.6 ± 2.7	30.7 ± 3	1516 ± 448	1576 ± 585	40 (88.9)	22 (71)
Boskabadi et al. (2024) [6]	80	80	NR	NR	32.2 ± 1.9	31.6 ± 2.2	1936 ± 581	1771 ± 580	59 (73.8)	58 (72.5)
Chen et al. (2020) [7]	30	30	17 (56.7)	17 (56.7)	31.1 ± 1.5	31 ± 1.5	2345 ± 225	2297 ± 217	NR	NR
Elbaz et al. (2021) [8]	45	62	22 (48.9)	41 (66.1)	32.2 ± 2.6	31.9 ± 2.8	1900 ± 600	1810 ± 590	26 (57.8)	35 (56.4)
Garib et al. (2021) [9]	35	35	18 (51.4)	20 (57.1)	28.1 ± 1.5	27.3 ± 1.8	—	NR	NR	NR
Gupta et al. (2020) [2]	29	29	18 (62.1)	18 (62)	30.1 ± 1.5	29.9 ± 1.7	1225 ± 281	1222 ± 322	23 (79.3)	24 (82.8)
Han et al. (2020) [10]	151	147	80 (53)	85 (57.9)	30.6 ± 1.6	30.8 ± 1.3	1427 ± 290	1419 ± 273	110 (72.8)	114 (77.6)
Jena et al. (2019) [11]	175	175	91 (520	77 (44)	31 ± 0.7	31 ± 0.7	1630 ± 156	1683 ± 135	106 (60.6)	111 (63.4)
Kaleem et al. (2023) [12]	36	36	23 (63.9)	22 (61.1)	28.5 ± 0.5	30.5 ± 0.55	NR	NR	NR	NR
Krajewski et al. (2022) [13]	34	60	19 (55.9)	31 (51.7)	29.9 ± 1.9	28.3 ± 1.8	1444 ± 433	1117 ± 387	20 (58.9)	27 (45)
Manuela et al. (2017) [14]	17	9	NR	NR	NR	NR	NR	NR	NR	NR
Mosayebi et al. (2017) [15]	27	26	16 (59.3)	11 (42.3)	32.6 ± 1.1	31.9 ± 1.5	1791 ± 553	1910 ± 433	14 (51.9)	15 (57.7)
Rallis et al. (2024) [16]	36	37	NR	NR	29.1 ± 2.2	28.8 ± 2.3	1219 ± 238	1195 ± 336	NR	NR
Sabzehei et al. (2022) [17]	56	56	37 (66.1)	38 (67.9)	29.7 ± 3	30.6 ± 3.4	1530 ± 507	1678 ± 543	33 (60)	38 (67.9)
Sayed et al. (2024) [18]	45	45	22 (48.9)	16 (35.6)	30.3 ± 2.1	30.4 ± 1.9	1145 ± 224	1149 ± 193	19 (42.2)	17 (37.8)
Wang et al. (2020) [3]	24	29	14 (58.3)	17 (58.6)	29.4 ± 1.6	28.7 ± 1.4	1240 ± 197	1140 ± 177	NR	NR
Zhao et al. (2020) [19]	48	45	NR	NR	NR	NR	NR	NR	NR	NR

*Note:* NR means not reported.

**Table 3:** Meta-regression analyses of mean gestational age and birth weight with primary outcomes. CI indicates confidence interval. *β*: regression coefficient; *p* value: considered significant with a value < 0.05.

**Variable**	**Studies**	**Moderator**	**β**	**95% CI**	**p** ** value**
Mortality	8	Gestational age (mean weeks)	−0.019	[−0.66, 0.62]	0.953
		Birth weight (mean)	0.002	[−0.0007, 0.004]	0.171
Bronchopulmonary dysplasia	12	Gestational age (mean weeks)	−0.12	[−0.48, 0.24]	0.519
		Birth weight (mean)	−0.0009	[−0.002, 0.0004]	0.204
Persistent ductus arteriosus	11	Gestational age (mean weeks)	0.08	[−0.15, 0.31]	0.491
		Birth weight (mean)	0.0004	[−0.0004, 0.0012]	0.352
Intraventricular hemorrhage	12	Gestational age (mean weeks)	0.2	[−0.08, 0.49]	0.168
		Birth weight (mean)	0.0003	[−0.001, 0.002]	0.643
Pneumothorax	8	Gestational age (mean weeks)	−0.155	[−0.94, 0.63]	0.697
		Birth weight (mean)	−0.0007	[−0.003, 0.001]	0.501

## Data Availability

The datasets and materials used during the course of this research are available upon reasonable request. Interested researchers can contact Flavio Veintemilla-Burgos (flavio.veintemilla@cu.ucsg.edu.ec) for access to the data and materials supporting the findings of this study.

## Supporting Information

Supporting Information Additional supporting information can be found online in the Supporting Information section. Figure S1: Funnel plot assessing publication bias of need for mechanical ventilation in the meta-analysis. The plot shows symmetrical distribution, suggesting no substantial small-study effects or publication bias. Outcomes were assessed using Egger's test, which also confirmed the absence of significant bias (*p* > 0.05). Figure S2: Funnel plot assessing publication bias for incidence of bronchopulmonary dysplasia in the meta-analysis. The plot shows symmetrical distribution, suggesting no substantial small-study effects or publication bias. Outcomes were assessed using Egger's test, which also confirmed the absence of significant bias (*p* > 0.05). Figure S3: Funnel plot assessing publication bias for incidence of intraventricular hemorrhage in the meta-analysis. The plot shows symmetrical distribution, suggesting no substantial small-study effects or publication bias. Outcomes were assessed using Egger's test, which also confirmed the absence of significant bias (*p* > 0.05). Figure S4: Funnel plot assessing publication bias for incidence of persistent ductus arteriosus in the meta-analysis. The plot shows symmetrical distribution, suggesting no substantial small-study effects or publication bias. Outcomes were assessed using Egger's test, which also confirmed the absence of significant bias (*p* > 0.05). Figure S5: Funnel plot assessing publication bias for incidence of pneumothorax in the meta-analysis. The plot shows symmetrical distribution, suggesting no substantial small-study effects or publication bias. Outcomes were assessed using Egger's test, which also confirmed the absence of significant bias (*p* > 0.05). Figure S6: Funnel plot assessing publication bias for days needing oxygen in the meta-analysis. The plot shows symmetrical distribution, suggesting no substantial small-study effects or publication bias. Outcomes were assessed using Egger's test, which also confirmed the absence of significant bias (*p* > 0.05). Table S1: Completed PRISMA 2020 checklist documenting adherence to the Preferred Reporting Items for Systematic Reviews and Meta-Analyses (PRISMA) guidelines. Each item includes references to the corresponding sections of the manuscript where the required information is reported. Table S2: Cochrane Risk of Bias 2 assessment. Table S3: Risk of bias assessment using the Newcastle–Ottawa Scale. Table S4: Assessment of certainty of evidence with the GRADE scale system.
