# Supplementary material for: Tiny Lungs, Big Decisions: A Meta-Analysis Comparing Minimally Invasive Surfactant Therapy Versus Intubation–Surfactant–Extubation in Preterm Neonates With Respiratory Distress Syndrome
Source: Int J Pediatr. 2025 Aug 14;2025:8045343. doi: 10.1155/ijpe/8045343 (PMC12370393; doi:10.1155/ijpe/8045343)
Supplement: Supporting Information — Additional supporting information can be found online in the Supporting Information section. Figure S1: Funnel plot assessing publication bias of need for mechanical ventilation in the meta-analysis. The plot shows symmetrical distribution, suggesting no substantial small-study effects or publication bias. Outcomes were assessed using Egger's test, which also confirmed the absence of significant bias (p > 0.05). Figure S2: Funnel plot assessing publication bias for incidence of bronchopulmonary dysplasia in the meta-analysis. The plot shows symmetrical distribution, suggesting no substantial small-study effects or publication bias. Outcomes were assessed using Egger's test, which also confirmed the absence of significant bias (p > 0.05). Figure S3: Funnel plot assessing publication bias for incidence of intraventricular hemorrhage in the meta-analysis. The plot shows symmetrical distribution, suggesting no substantial small-study effects or publication bias. Outcomes were assessed using Egger's test, which also confirmed the absence of significant bias (p > 0.05). Figure S4: Funnel plot assessing publication bias for incidence of persistent ductus arteriosus in the meta-analysis. The plot shows symmetrical distribution, suggesting no substantial small-study effects or publication bias. Outcomes were assessed using Egger's test, which also confirmed the absence of significant bias (p > 0.05). Figure S5: Funnel plot assessing publication bias for incidence of pneumothorax in the meta-analysis. The plot shows symmetrical distribution, suggesting no substantial small-study effects or publication bias. Outcomes were assessed using Egger's test, which also confirmed the absence of significant bias (p > 0.05). Figure S6: Funnel plot assessing publication bias for days needing oxygen in the meta-analysis. The plot shows symmetrical distribution, suggesting no substantial small-study effects or publication bias. Outcomes were assessed using Egger's test, which also confirmed [file 8045343.f1.zip › PRISMA_2020_checklist_VeintemillaBurgos.docx]

| **Section and Topic** | **Item #** | **Checklist item** | **Location where item is reported** |
| --- | --- | --- | --- |
| **TITLE** | | |  |
| Title | 1 | Identify the report as a systematic review. | ✅Title page |
| **ABSTRACT** | | |  |
| Abstract | 2 | See the PRISMA 2020 for Abstracts checklist. | ✅Page 1 |
| **INTRODUCTION** | | |  |
| Rationale | 3 | Describe the rationale for the review in the context of existing knowledge. | ✅ Page 2 Lines 36 - 57 |
| Objectives | 4 | Provide an explicit statement of the objective(s) or question(s) the review addresses. | ✅Page 2  lines 57- 61 |
| **METHODS** | | |  |
| Eligibility criteria | 5 | Specify the inclusion and exclusion criteria for the review and how studies were grouped for the syntheses. | ✅Page 2 - 3  Lines 76 - 88 |
| Information sources | 6 | Specify all databases, registers, websites, organisations, reference lists and other sources searched or consulted to identify studies. Specify the date when each source was last searched or consulted. | ✅ Page 2  Lines 68 - 72 |
| Search strategy | 7 | Present the full search strategies for all databases, registers and websites, including any filters and limits used. | ✅ Page 2  Lines 68 - 72 |
| Selection process | 8 | Specify the methods used to decide whether a study met the inclusion criteria of the review, including how many reviewers screened each record and each report retrieved, whether they worked independently, and if applicable, details of automation tools used in the process. | ✅ Page 3  Lines 92-102 |
| Data collection process | 9 | Specify the methods used to collect data from reports, including how many reviewers collected data from each report, whether they worked independently, any processes for obtaining or confirming data from study investigators, and if applicable, details of automation tools used in the process. | ✅ Page 3  Lines 92-102 |
| Data items | 10a | List and define all outcomes for which data were sought. Specify whether all results that were compatible with each outcome domain in each study were sought (e.g. for all measures, time points, analyses), and if not, the methods used to decide which results to collect. | ✅ Page 3  Lines 93-102 |
|  | 10b | List and define all other variables for which data were sought (e.g. participant and intervention characteristics, funding sources). Describe any assumptions made about any missing or unclear information. | ✅ Page 3  Lines 116-126 |
| Study risk of bias assessment | 11 | Specify the methods used to assess risk of bias in the included studies, including details of the tool(s) used, how many reviewers assessed each study and whether they worked independently, and if applicable, details of automation tools used in the process. | ✅ Page 3  Lines 108-112 ; 135-139 |
| Effect measures | 12 | Specify for each outcome the effect measure(s) (e.g. risk ratio, mean difference) used in the synthesis or presentation of results. | ✅ Page 4  Lines 130-135 |
| Synthesis methods | 13a | Describe the processes used to decide which studies were eligible for each synthesis (e.g. tabulating the study intervention characteristics and comparing against the planned groups for each synthesis (item #5)). | ✅ Page 3  Lines 97-108 |
|  | 13b | Describe any methods required to prepare the data for presentation or synthesis, such as handling of missing summary statistics, or data conversions. | ✅ Page 3  Lines 92-102 |
|  | 13c | Describe any methods used to tabulate or visually display results of individual studies and syntheses. | ✅ Page 3  Lines 92-102 |
|  | 13d | Describe any methods used to synthesize results and provide a rationale for the choice(s). If meta-analysis was performed, describe the model(s), method(s) to identify the presence and extent of statistical heterogeneity, and software package(s) used. | ✅ Page 3-4  Lines 130-139 |
|  | 13e | Describe any methods used to explore possible causes of heterogeneity among study results (e.g. subgroup analysis, meta-regression). | ✅ Page 3-4  Lines 133-135 |
|  | 13f | Describe any sensitivity analyses conducted to assess robustness of the synthesized results. | ✅ Page 3-4  Lines 130-139 |
| Reporting bias assessment | 14 | Describe any methods used to assess risk of bias due to missing results in a synthesis (arising from reporting biases). | ✅ Page 3-4  Lines 135-139 |
| Certainty assessment | 15 | Describe any methods used to assess certainty (or confidence) in the body of evidence for an outcome. | ✅ Page 5  Lines 204-209 |
| **RESULTS** | | |  |
| Study selection | 16a | Describe the results of the search and selection process, from the number of records identified in the search to the number of studies included in the review, ideally using a flow diagram. | ✅ Figure #1 |
|  | 16b | Cite studies that might appear to meet the inclusion criteria, but which were excluded, and explain why they were excluded. | ✅ Figure #1 |
| Study characteristics | 17 | Cite each included study and present its characteristics. | ✅ Table #1 |
| Risk of bias in studies | 18 | Present assessments of risk of bias for each included study. | ✅ Page 5  Lines 194-200 |
| Results of individual studies | 19 | For all outcomes, present, for each study: (a) summary statistics for each group (where appropriate) and (b) an effect estimate and its precision (e.g. confidence/credible interval), ideally using structured tables or plots. | ✅ Figures #2 - #7  Lines 154-170 |
| Results of syntheses | 20a | For each synthesis, briefly summarise the characteristics and risk of bias among contributing studies. | ✅ Page 5  Lines 194-200 |
|  | 20b | Present results of all statistical syntheses conducted. If meta-analysis was done, present for each the summary estimate and its precision (e.g. confidence/credible interval) and measures of statistical heterogeneity. If comparing groups, describe the direction of the effect. | ✅ Page 4-5  Lines 184-192 |
|  | 20c | Present results of all investigations of possible causes of heterogeneity among study results. | ✅ Page 5  Lines 194-200 |
|  | 20d | Present results of all sensitivity analyses conducted to assess the robustness of the synthesized results. | ✅ Page 5  Lines 194-200 |
| Reporting biases | 21 | Present assessments of risk of bias due to missing results (arising from reporting biases) for each synthesis assessed. | ✅ Page 5  Lines 194-200 |
| Certainty of evidence | 22 | Present assessments of certainty (or confidence) in the body of evidence for each outcome assessed. | ✅ Page 5  Lines 204-209 |
| **DISCUSSION** | | |  |
| Discussion | 23a | Provide a general interpretation of the results in the context of other evidence. | ✅ Page 5  Lines 213-215 |
|  | 23b | Discuss any limitations of the evidence included in the review. | ✅ Page 6  Lines 243-257 |
|  | 23c | Discuss any limitations of the review processes used. | ✅ Page 6  Lines 243-257 |
|  | 23d | Discuss implications of the results for practice, policy, and future research. | ✅ Page 6  Lines 261-266 |
| **OTHER INFORMATION** | | |  |
| Registration and protocol | 24a | Provide registration information for the review, including register name and registration number, or state that the review was not registered. | ✅ Page 16  Line 359 |
|  | 24b | Indicate where the review protocol can be accessed, or state that a protocol was not prepared. | ✅ Page 3  Lines 104-106 |
|  | 24c | Describe and explain any amendments to information provided at registration or in the protocol. |  |
| Support | 25 | Describe sources of financial or non-financial support for the review, and the role of the funders or sponsors in the review. | ✅ Page 16 |
| Competing interests | 26 | Declare any competing interests of review authors. | ✅ Page 16 |
| Availability of data, code and other materials | 27 | Report which of the following are publicly available and where they can be found: template data collection forms; data extracted from included studies; data used for all analyses; analytic code; any other materials used in the review. | ✅ Page 3  Lines 14-106  &  Page 16  Lines 352-355 |

*From:*  Page MJ, McKenzie JE, Bossuyt PM, Boutron I, Hoffmann TC, Mulrow CD, et al. The PRISMA 2020 statement: an updated guideline for reporting systematic reviews. BMJ 2021;372:n71. doi: 10.1136/bmj.n71
