# Supplementary material for: Tiny Lungs, Big Decisions: A Meta-Analysis Comparing Minimally Invasive Surfactant Therapy Versus Intubation–Surfactant–Extubation in Preterm Neonates With Respiratory Distress Syndrome
Source: Int J Pediatr. 2025 Aug 14;2025:8045343. doi: 10.1155/ijpe/8045343 (PMC12370393; doi:10.1155/ijpe/8045343)
Supplement: Supporting Information — Additional supporting information can be found online in the Supporting Information section. Figure S1: Funnel plot assessing publication bias of need for mechanical ventilation in the meta-analysis. The plot shows symmetrical distribution, suggesting no substantial small-study effects or publication bias. Outcomes were assessed using Egger's test, which also confirmed the absence of significant bias (p > 0.05). Figure S2: Funnel plot assessing publication bias for incidence of bronchopulmonary dysplasia in the meta-analysis. The plot shows symmetrical distribution, suggesting no substantial small-study effects or publication bias. Outcomes were assessed using Egger's test, which also confirmed the absence of significant bias (p > 0.05). Figure S3: Funnel plot assessing publication bias for incidence of intraventricular hemorrhage in the meta-analysis. The plot shows symmetrical distribution, suggesting no substantial small-study effects or publication bias. Outcomes were assessed using Egger's test, which also confirmed the absence of significant bias (p > 0.05). Figure S4: Funnel plot assessing publication bias for incidence of persistent ductus arteriosus in the meta-analysis. The plot shows symmetrical distribution, suggesting no substantial small-study effects or publication bias. Outcomes were assessed using Egger's test, which also confirmed the absence of significant bias (p > 0.05). Figure S5: Funnel plot assessing publication bias for incidence of pneumothorax in the meta-analysis. The plot shows symmetrical distribution, suggesting no substantial small-study effects or publication bias. Outcomes were assessed using Egger's test, which also confirmed the absence of significant bias (p > 0.05). Figure S6: Funnel plot assessing publication bias for days needing oxygen in the meta-analysis. The plot shows symmetrical distribution, suggesting no substantial small-study effects or publication bias. Outcomes were assessed using Egger's test, which also confirmed [file 8045343.f1.zip › Supplementary_VeintemillaBurgos.docx]

**Title**

Comparing Outcomes of Minimally Invasive Surfactant Therapy vs. Intubation-Surfactant-Extubation in Preterm Neonates with Neonatal Respiratory Distress Syndrome: A Meta-Analysis

**Authors**:

Flavio Veintemilla-Burgos, MD 1,2, Geovanna Minchalo-Ochoa, MD 1,2, Sebastian Balda, MD 1,2, Ivo Diaz-Djevoich, MD 1,2, Rodolfo Kronfle, MD 1,2, Matias Panchana-Lascano, MD 1,2 , Thomas Leone-Berry, MD 1,2

**Affiliations**:

1 Universidad Católica Santiago de Guayaquil 09014671, Ecuador

2 Summa Veritas Medical Research. Hospital Clinica Kennedy. Guayaquil 090510, Ecuador

**CorrespondingAuthor**: Flavio Veintemilla-Burgos, MD. Summa Veritas Medical Research. Hospital Clinica Kennedy, Office 31-A. Av. del Periodista y Callejón 11-A, N.O Kennedy. Guayaquil 090510, Ecuador, Tel +593 985067520. Email: [flavioveintemilla@gmail.com](mailto:flavioveintemilla@gmail.com)

**Supplementary Material**

**
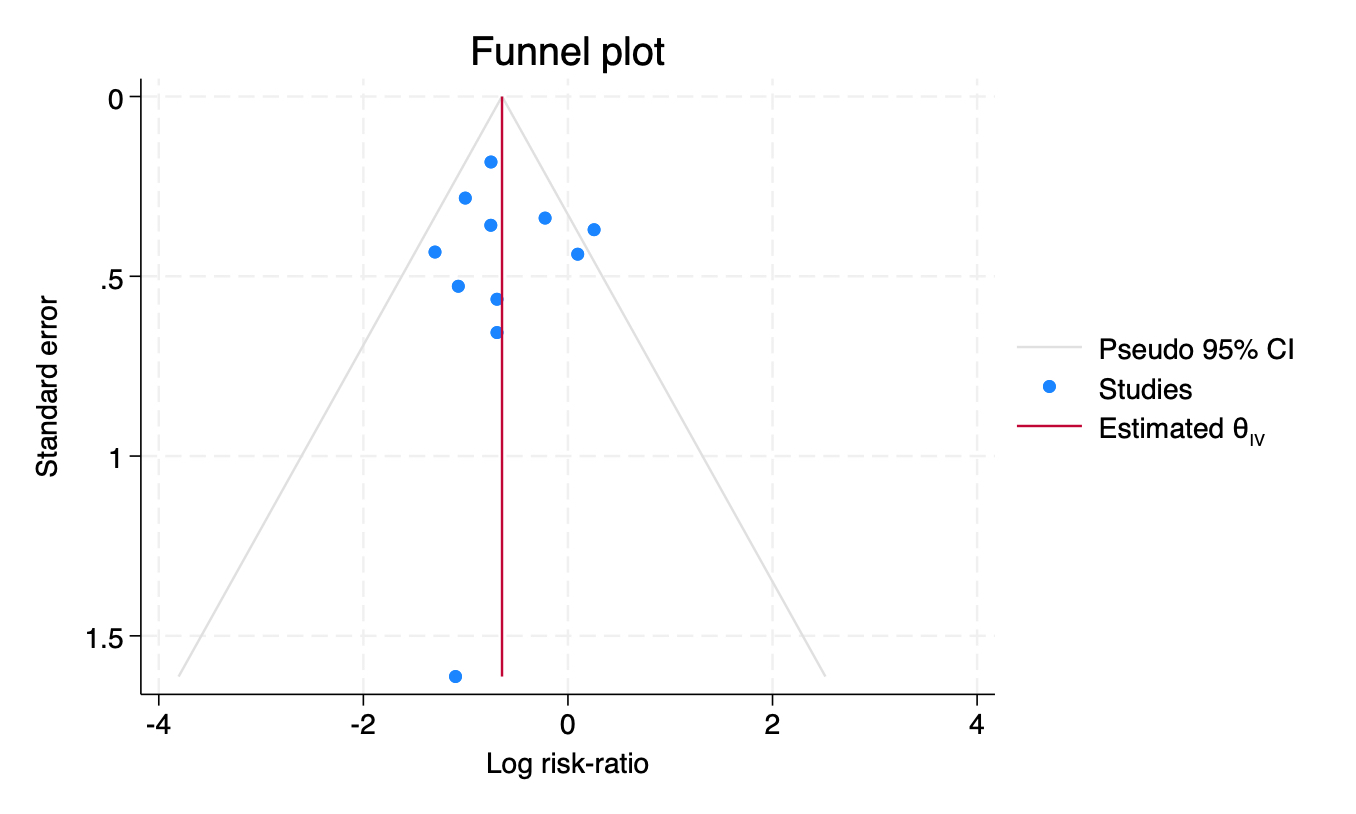
**

Figure S1. Funnel plot assessing publication bias of need for mechanical ventilation in the meta-analysis. The plot shows symmetrical distribution, suggesting no substantial small-study effects or publication bias. Outcomes were assessed using Egger’s test, which also confirmed the absence of significant bias (p > 0.05).


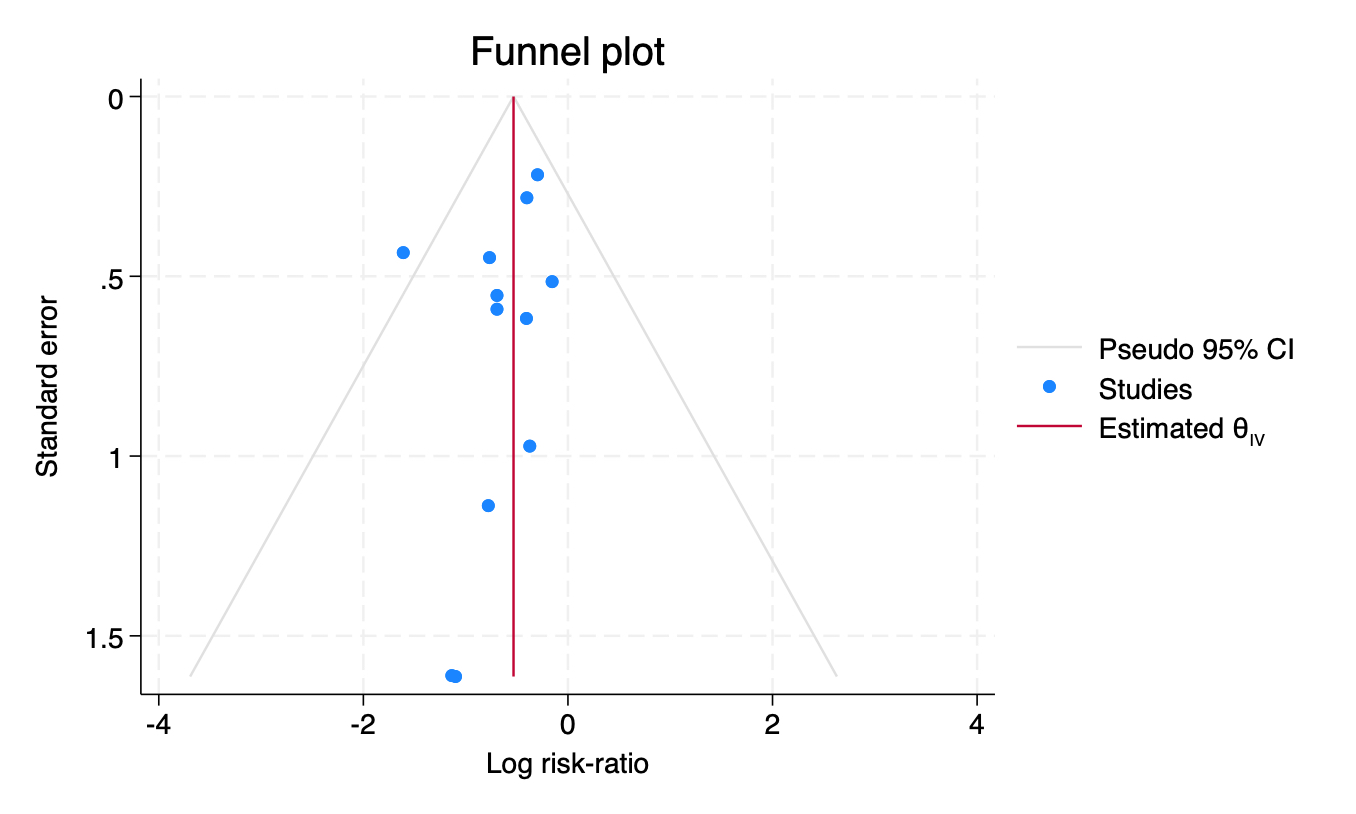


Figure S2. Funnel plot assessing publication bias for incidence of bronchopulmonary dysplasia in the meta-analysis. The plot shows symmetrical distribution, suggesting no substantial small-study effects or publication bias. Outcomes were assessed using Egger’s test, which also confirmed the absence of significant bias (p > 0.05).


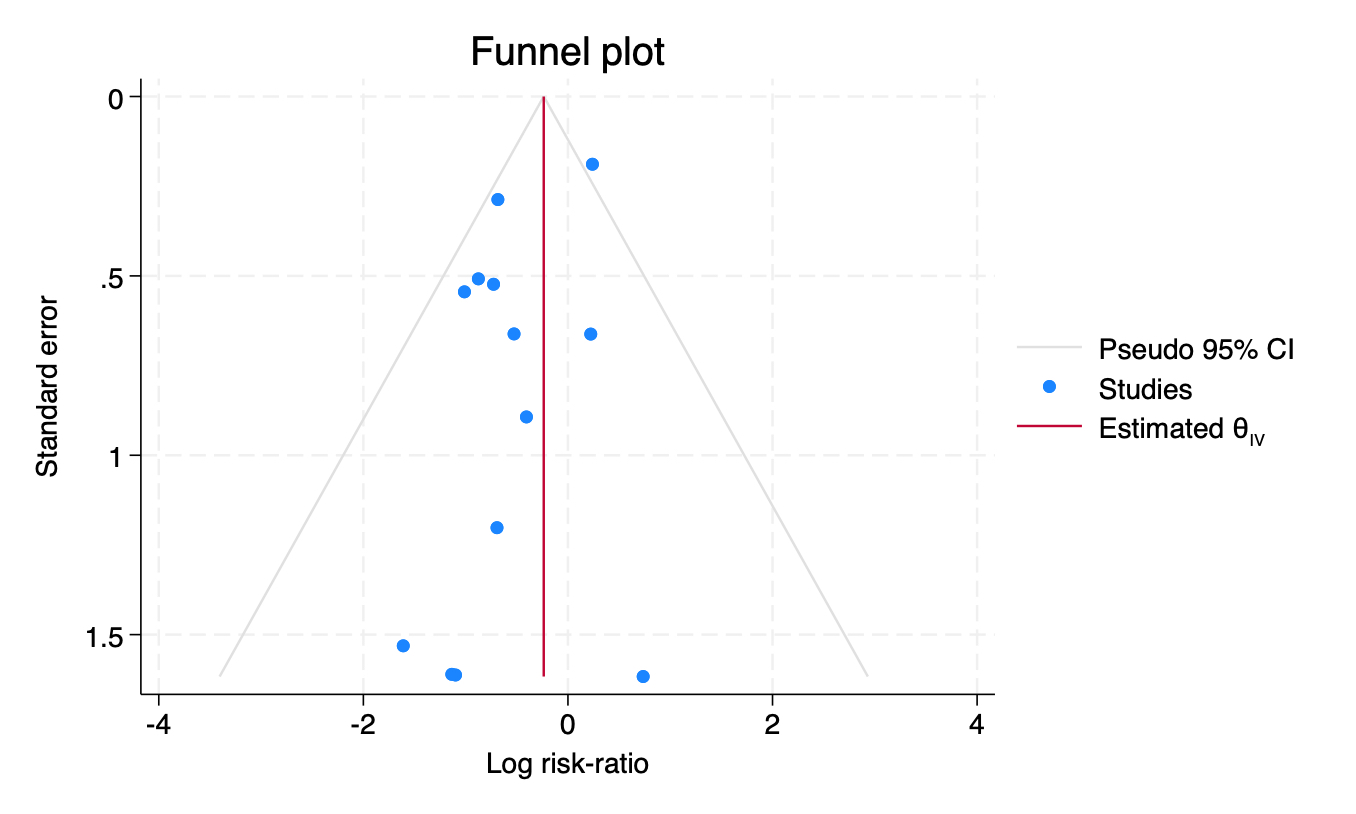


Figure S3. Funnel plot assessing publication bias for incidence of intraventricular hemorrhage in the meta-analysis. The plot shows symmetrical distribution, suggesting no substantial small-study effects or publication bias. Outcomes were assessed using Egger’s test, which also confirmed the absence of significant bias (p > 0.05).


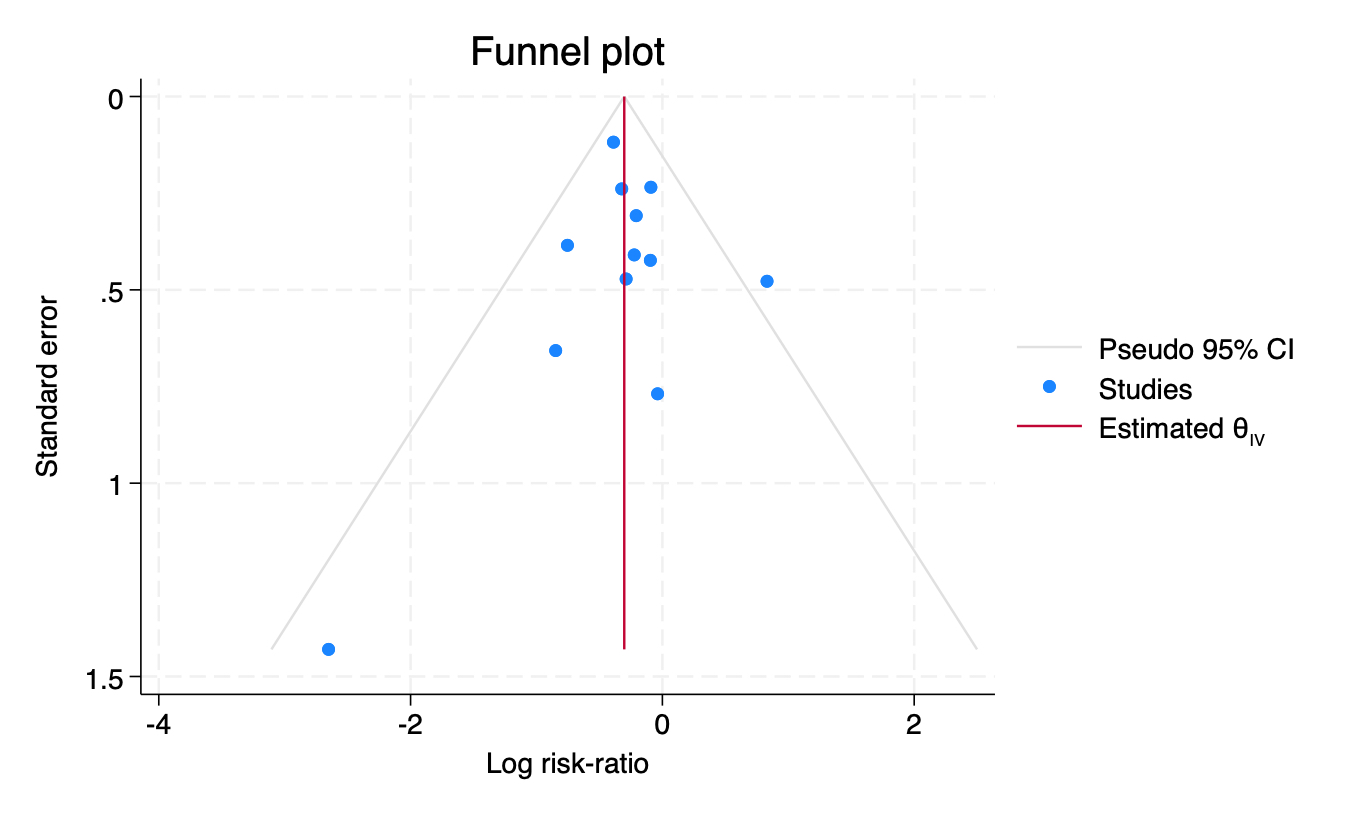


Figure S4. Funnel plot assessing publication bias for incidence of persistent ductus arteriosus in the meta-analysis. The plot shows symmetrical distribution, suggesting no substantial small-study effects or publication bias. Outcomes were assessed using Egger’s test, which also confirmed the absence of significant bias (p > 0.05).


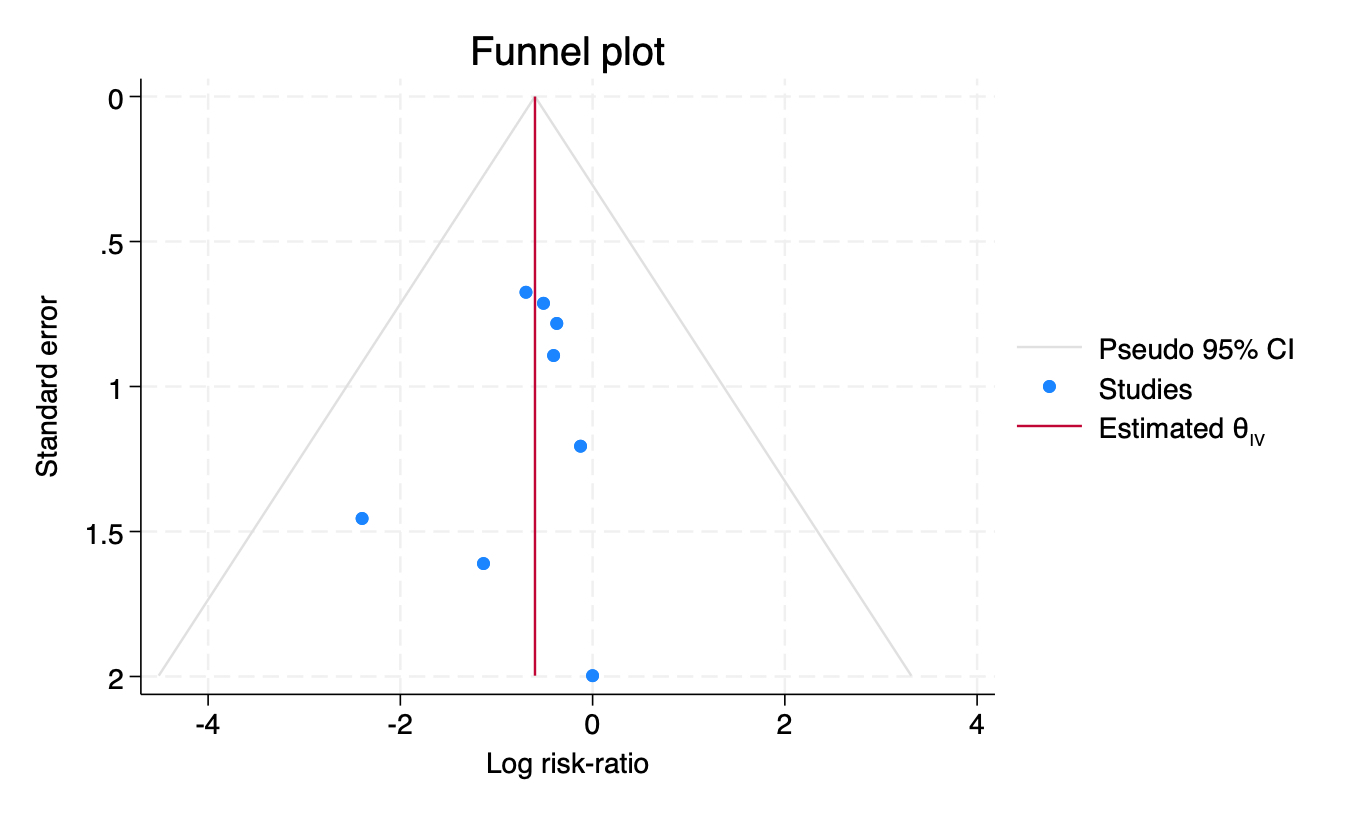


Figure S5. Funnel plot assessing publication bias for incidence of pneumothorax in the meta-analysis. The plot shows symmetrical distribution, suggesting no substantial small-study effects or publication bias. Outcomes were assessed using Egger’s test, which also confirmed the absence of significant bias (p > 0.05).


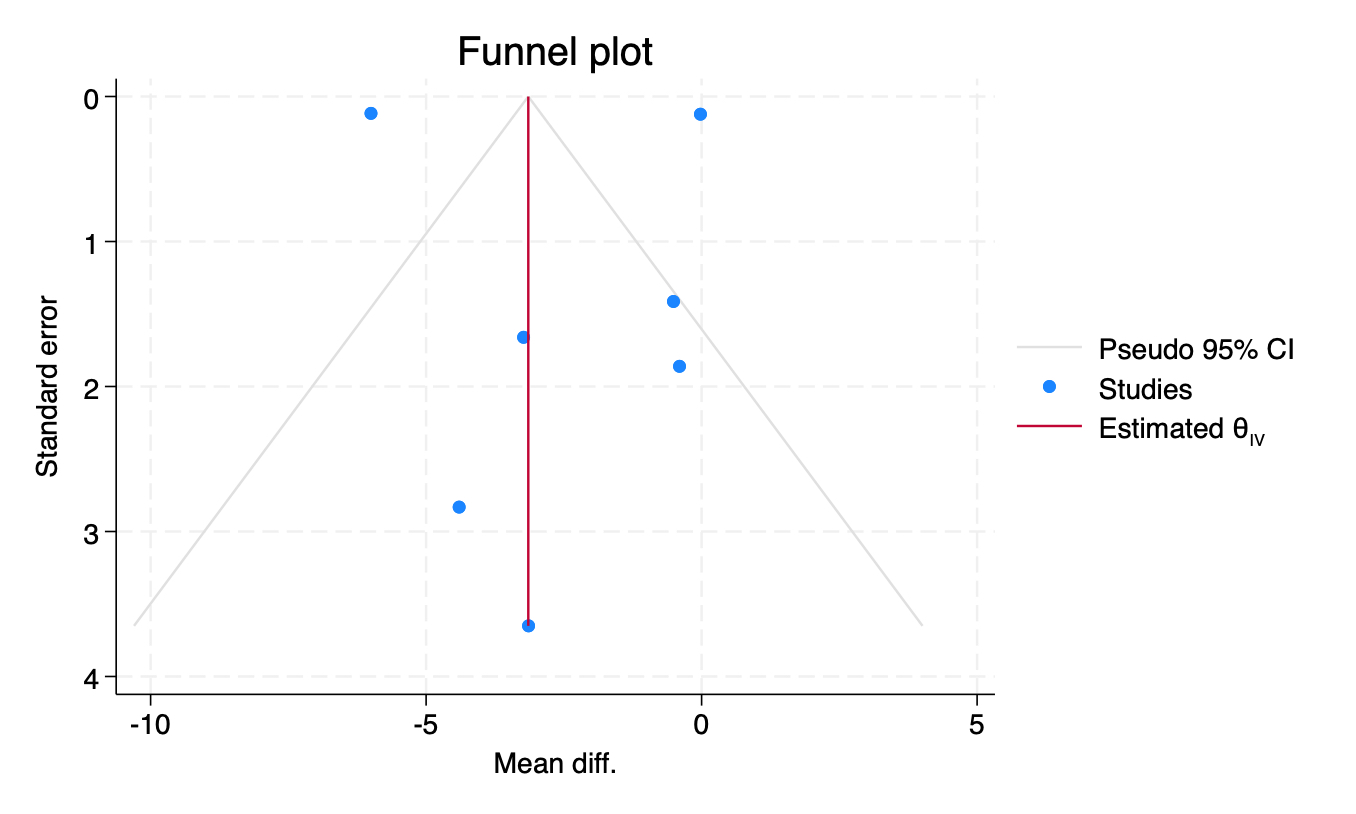


Figure S6. Funnel plot assessing publication bias for days needing oxygen in the meta-analysis. The plot shows symmetrical distribution, suggesting no substantial small-study effects or publication bias. Outcomes were assessed using Egger’s test, which also confirmed the absence of significant bias (p > 0.05).

| **Cochrane Risk of Bias 2 assessment** | Boskabadi et al, 2024 | Chen et al, 2020 | Garib et al, 2021 | Gupta et al, 2020 | Han et al, 2020 | Jena et al, 2019 | Kaleem et al, 2023 | Manuela et al, 2017 | Mosayebi et al, 2017 | Sabzehei et al, 2022 | Sayed et al, 2024 | Zhao et al, 2020 |
| --- | --- | --- | --- | --- | --- | --- | --- | --- | --- | --- | --- | --- |
| Bias arising from the randomization process | **+** | **+** | **+** | **+** | **+** | **+** | **+** | **!** | **+** | **+** | **+** | **+** |
| Bias due to deviations from intended interventions | **+** | **?** | **+** | **?** | **?** | **?** | **?** | **?** | **?** | **+** | **?** | **?** |
| Bias due to missing outcome data | **+** | **+** | **+** | **+** | **+** | **+** | **+** | **?** | **+** | **+** | **+** | **+** |
| Bias in measurement of the outcome | **+** | **?** | **+** | **!** | **+** | **+** | **+** | **+** | **+** | **+** | **+** | **+** |
| Bias in selection of the reported result | **+** | **+** | **+** | **+** | **+** | **+** | **?** | **?** | **+** | **+** | **?** | **+** |

Table S2. Cochrane Risk of Bias 2 assessment

| **Study ID** | **Selection** | | | | **Comparability** | | **Outcome** | | | **Score** |
| --- | --- | --- | --- | --- | --- | --- | --- | --- | --- | --- |
|  | **Representativeness of the exposed** | **Selection of Non-Exposed** | **Ascertainment of Exposure** | **Outcome of Interest Not Present at Start of Study** | **Main Factor** | **Aditional Factor** | **Assessment** | **Follow-up Lenght** | **Adequacy of follow-up** |  |
| Aguar et al, 2014 | ***** | ***** | ***** | ***** | ***** | **0** | ***** | ***** | ***** | **8** |
| Elbaz et al, 2021 | ***** | ***** | ***** | **0** | ***** | ***** | ***** | ***** | **0** | **7** |
| Krajewski et al, 2022 | ***** | ***** | ***** | ***** | ***** | ***** | ***** | ***** | ***** | **9** |
| Rallis et al, 2024 | ***** | ***** | ***** | ***** | ***** | ***** | ***** | ***** | ***** | **9** |
| Wang et al, 2020 | ***** | ***** | ***** | **0** | ***** | **0** | ***** | **0** | ***** | **6** |

Table S3. Risk of Bias Assessment using the NewCastle-Ottawa Scale

| **Outcomes** | **Effect Estimates (RR or MD)** | **No. ofStudies** | **CertaintyofEvidence (GRADE)** | **Justification** |
| --- | --- | --- | --- | --- |
| **Mortality** | RR 0.62 (95% CI 0.38–1.01), p=0.05 | 17 | Moderate | Downgraded for imprecision (borderline significance) |
| **BronchopulmonaryDysplasia** | RR 0.59 (95% CI 0.45–0.76), p<0.001 | 12 | High | No downgrades: consistent and precise |
| **Intraventricular Hemorrhage** | RR 0.66 (95% CI 0.48–0.92), p=0.02 | 10 | Moderate | Downgraded for inconsistency (sensitive to one study) |
| **Patent Ductus Arteriosus** | RR 0.75 (95% CI 0.61–0.93), p=0.01 | 9 | Moderate | Downgradedforinconsistency |
| **Pneumothorax** | RR 0.54 (95% CI 0.34–0.87), p=0.02 | 8 | High | No downgrades: consistent, precise, no publicationbias |
| **PulmonaryHemorrhage** | RR 0.69 (95% CI 0.38–1.25), p=0.18 | 6 | Low | Downgraded for imprecision and inconsistency |
| **SurfactantReflux** | RR 1.47 (95% CI 0.71–3.03), p=0.22 | 5 | Low | Wide CI, imprecise |
| **DaysofOxygen Use** | MD -2.45 days (95% CI -4.78 to -0.12), p=0.04 | 6 | Moderate | Downgraded for imprecision (sensitive to one study) |
| **NeedforMechanicalVentilation** | RR 0.54 (95% CI 0.39–0.75), p=0.002 | 10 | High | Consistent, precise, robust |
| **DurationofMechanicalVentilation** | MD -1.83 (95% CI -6.15 to 2.50), p=0.34 | 5 | Low | Not significant; downgraded for imprecision |
| **NICU StayDuration** | MD -4.08 (95% CI -8.89 to 0.83), p=0.09 | 5 | Low | Downgraded for imprecision and inconsistency |

Table S4. Assessment of certainty of evidence with the GRADE scale system.
